# Expression levels of HMGA2 and CD9 and its clinicopathological significances in the benign and malignant lesions of the gallbladder

**DOI:** 10.1186/1477-7819-10-92

**Published:** 2012-05-21

**Authors:** Qiong Zou, Li Xiong, Zhulin Yang, Fang Lv, Leping Yang, Xiongying Miao

**Affiliations:** 1 Department of Pathology, Third Xiangya Hospital, Central South University, Changsha, Hunan, 410013, China; 2 Research Laboratory of Hepatobiliary Diseases, Second Xiangya Hospital, Central South University, Changsha, Hunan, 410011, China

**Keywords:** Gallbladder neoplasms, Gallbladder polyp, Chronic cholecystitis, High mobility group A2, Mobility related protein-1/CD9, Immunohistochemsitry

## Abstract

**Background:**

The objective of this study was to investigate CD9 and HMGA2 expression and its clinicopathological significance in benign and malignant lesion tissues of the gallbladder.

**Methods:**

The resected specimens of 108 cases of gallbladder adenocarcinoma, 46 cases of adjacent tissue, 15 cases of polyps and 35 cases of chronic cholecystitis were made into conventional paraffin-embedded sections, using the method of EnVision immunohistochemistry to stain HMGA2 and CD9.

**Results:**

HMGA2 expression of gallbladder adenocarcinoma was significantly higher than that of adenocarcinoma adjacent tissues (= 16.13, *P* <0.01), polyps (= 8.19, *P* <0.01) and chronic cholecystitis (= 21.41, *P* <0.01); but CD9 expression was the opposite (*P* <0.05 or *P* <0.01). The positive rate of HMGA2 expression from the cases that had well-differentiated adenocarcinoma, with the largest tumor diameter <2 cm, and without lymph node metastasis, and that did not invade the surrounding tissue was significantly lower than that of HMGA2 expression from the cases that had poorly differentiated adenocarcinoma, with the largest tumor diameter ≥2 cm, lymph node metastasis, and that invaded the surrounding tissues (*P* <0.05 or *P* <0.01). The positive rate of CD9 expression from the cases that had well-differentiated adenocarcinoma, with the largest tumor diameter <2 cm, and without lymph node metastasis, and that did not invade the surrounding tissue was significantly higher than that of CD9 expression from the cases that had poorly differentiated adenocarcinoma, with the largest tumor diameter ≥2 cm, lymph node metastasis, and which invaded the surrounding tissues (*P* <0.05 or *P* <0.01). The Kaplan-Meier survival analysis showed that after surgery, the survival period of HMGA2 expression-positive cases was significantly lower than that of HMGA2 expression- negative cases (*P* = 0.020), but the survival period of CD9 expression-positive cases was significantly higher than that of cases with CD9 expression-negative (*P* = 0.019). Cox multivariate regression analysis showed that the HMGA2 positive expression and/or CD9 negative expression was an important indicator reflecting the poor prognosis of gallbladder cancer.

**Conclusion:**

The expression of HMGA2 and/or CD9 might be closely related to the carcinogenesis, clinical biological behaviors and prognosis of gallbladder adenocarcinoma.

## Background

Gallbladder cancer is the main malignancy occurring predominantly in older women. It accounts for nearly two-thirds of the biliary tract cancers, making it the most common primary biliary cancer and the fifth most common cancer of the gastrointestinal tract [1,2]. More than 85% of gallbladder cancers belong to adenocarcinomas, and the rest, approximately 15%, are squamous, adenosquamous or undifferentiated carcinomas.

High mobility group protein A2 (high mobility group A2, HMGA2) is a recently discovered non-histone chromatin protein, which is closely related to tumorigenesis, invasion and metastasis of tumors, which have high expression in epithelial or interstitial malignant tumors, and have high extent and levels of expression, are dependent on the metastasis of malignant tumors, and have poor prognosis [3-8].

The CD9 protein, also known as migration − 1 (mobility related protein-1, MRP1), belongs to the transmembrane 4 superfamily (TM4SF), and is a glycoproteinthat could inhibit cell movement. Recent studies showed that the CD9 expression level was closely related to tumor progression, metastasis and invasion of tumors and that tumors with low CD9 expression were strongly prone to metastasis and invasion [9-15].

In this study, by application of EnVision immunohistochemistry, we investigated HMGA2 and CD9 expression levels in benign and malignant lesion tissues of the gallbladder and studied the clinicopathological significance of their expression in the prognosis of gallbladder tumors.

## Methods

### Clinical data

From June 1996 to June 2006, 108 cases of surgical specimens of gallbladder adenocarcinoma were collected in the Second Xiangya Hospital, Xiangya Hospital and Hunan Provincial People's Hospital. These included 31 male (28.7%) and 77 female cases (71.3%), with age ranges from 35 to 70 years (the mean age was 52.6 ± 11.2 years). Pathological types included 9 cases of adenoma canceration (8.2%, 7 well-differentiated cases and 2 moderately differentiated cases), 29 cases of well differentiated adenocarcinoma (26.9%), 29 cases of moderately differentiated adenocarcinoma (26.9%), 30 cases of poorly differentiated adenocarcinoma (27.8%), and 11 cases of mucinous adenocarcinoma (10.2%). Among 108 cases of gallbladder adenocarcinoma, there were 59 cases with tissues and organs surrounding the gallbladder damaged (54.6%); 59 cases with regional lymph node metastasis (54.6%); 58 cases with gallbladder stones (53.7%); 34 cases of radical surgical resection (31.5%), 48 cases of palliative surgery (44.4%), and 26 cases with disease inspection samples taken due to widespread metastasis (24.1%). The survival analysis data of 67 cases of 108 cases of gallbladder adenocarcinoma were obtained by means of letters and telephones. These included 20 cases with a survival period of ≥1 year and 47 cases with a survival period of <1 year. In 46 cases of gallbladder adenocarcinoma cancer tissues (from cancer ≥3 mm), selected from 108 cases gallbladder adenocarcinoma, according to the gallbladder epithelial dysplasia diagnostic criteria provided by Yamagiwa [11], there were 10 normal cases, 10 cases of mild dysplasia, 12 cases of moderate dysplasia and 14 cases of severe dysplasia. From June 1996 to June 2006, 15 polyps resected gallbladder specimens were collected in the Second Xiangya Hospital, including those of 5 males (33.3%) and 10 females (66.7%)( age ranges were 42 to 60 years with a mean age of 50.8 ± 9.6 years. For these cases, the maximum polyp diameter range was 8 to 15 mm. These specimens were all confirmed by pathologic examination to be adenomatous polyps. Of these, 10 cases were from normal to mild gallbladder epithelial dysplasia, and 5 cases were from moderate to severe dysplasia. Fifteen cases of simple chronic cholecystitis and 20 cases of chronic cholecystitis with cholelithiasis were selected from the Second Xiangya Hospital, as the chronic cholecystitis control group, including 15 male cases (42.9%) and 20 female (57.1%), with an age range of 31 to 58 years, with a mean age of (43.2 ± 12.4) years. The pathologic examination confirmed that in the 35 cases, there were 11 cases with normal gallbladder mucosa, 12 cases of mild dysplasia, 7 cases of moderate dysplasia and 5 cases of severe dysplasia. All the specimens were fixed in 4% formaldehyde and made into routine paraffin-embedded sections, and sliced 4 μm thick.

### Reagents

The mouse anti-human HMGA2 monoclonal antibodies were purchased from Abcam Ltd., (Cambridge, UK). The CD9 monoclonal antibodies were purchased from Dako Laboratories,(Carpinteria, CA, USA).

The EnVisionTM staining kits were purchased from Gene Company, (Basel, Switzerland).

## Methods

**Figure 1:**
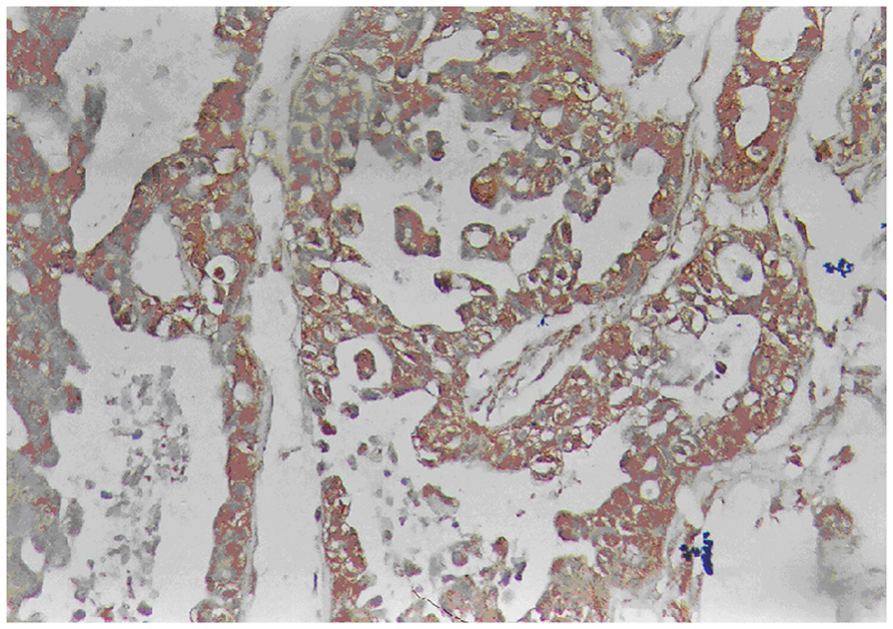
HMGA2-positive expression in the moderately differentiated adenocarcinoma of gallbladder by means of EnVision immunohistochemistry.

**Figure 2:**
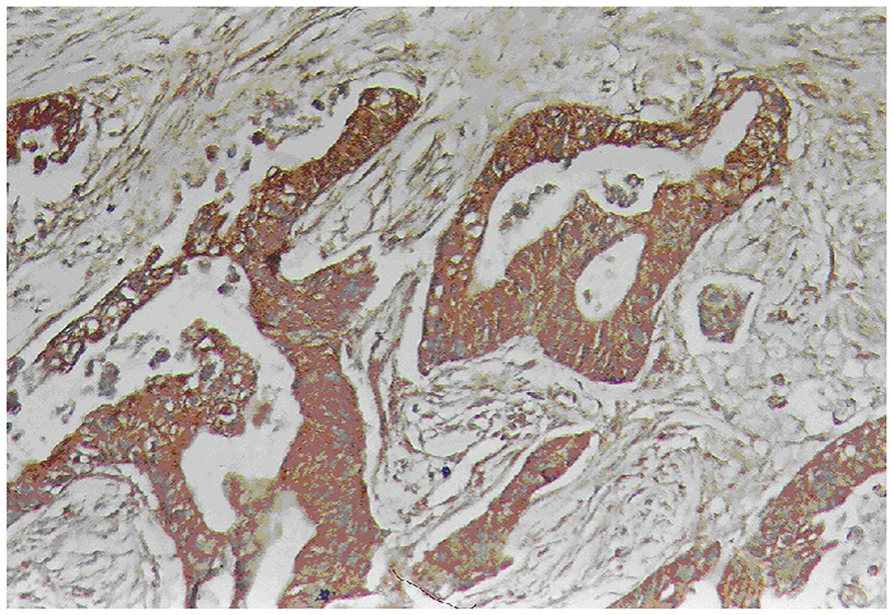
CD9-positive expression in the well-differentiated adenocarcinoma of gallbladder by means of EnVision immunohistochemistry.

**Figure 3:**
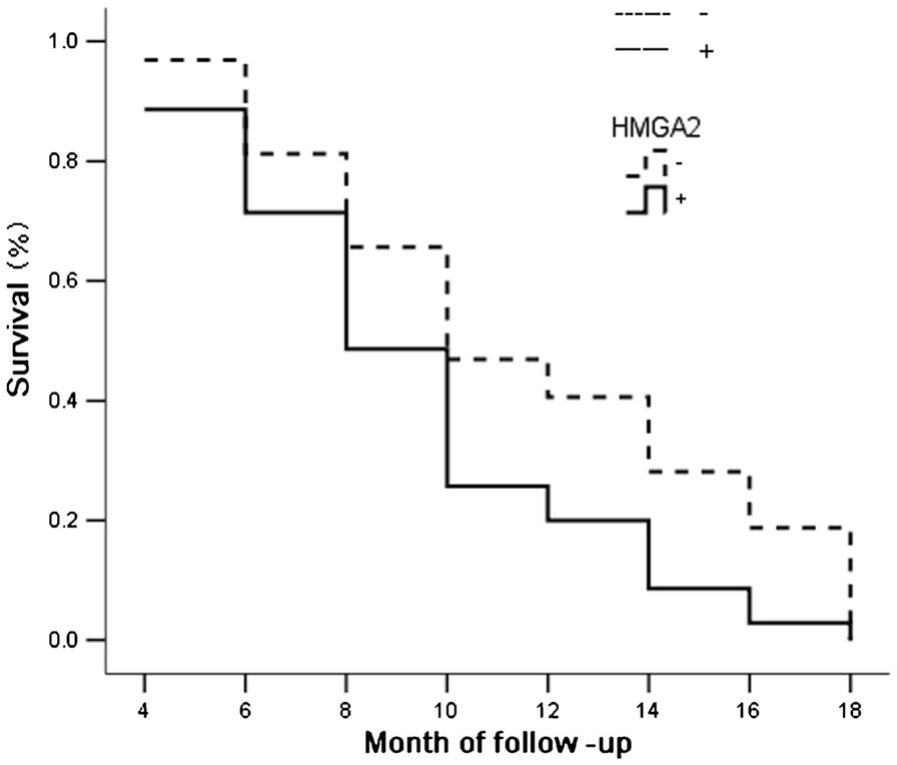
The survival curve of patients with HMGA2-positive or negative expression.

**Figure 4:**
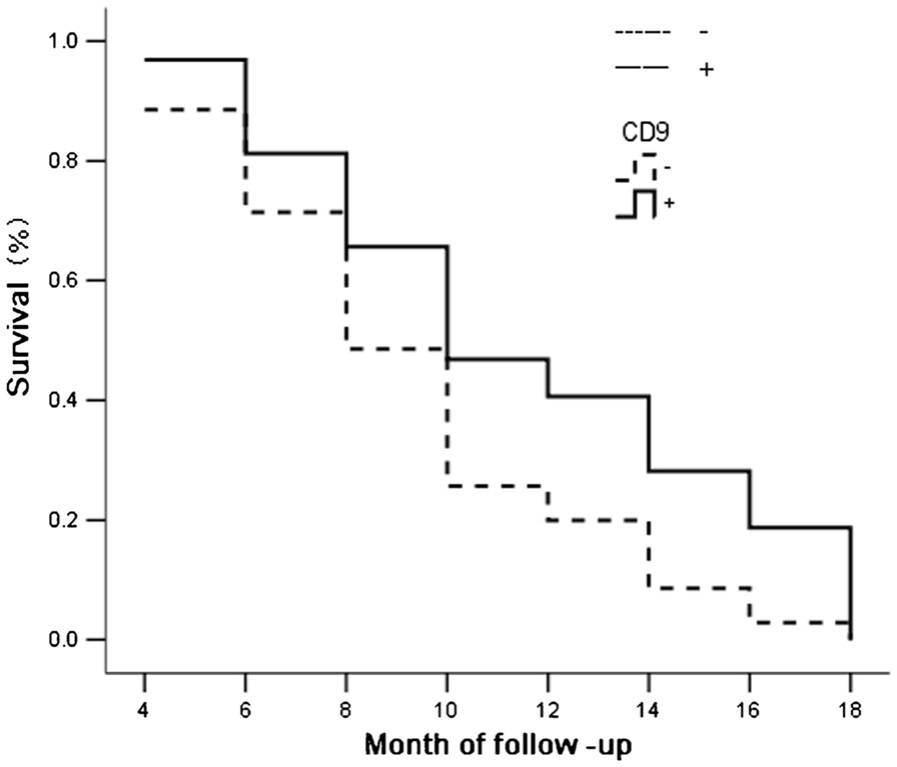
The survival curve of patients with CD9-positive or negative expression.

The EnVision two-step method was used to stain CD9 and HMGA2, strictly according to the manufacturer’s directions, as follows: Dewaxing to water washing → 3% H_2_O_2_ methanol solution for 10 minutes → trypsin for 15 minutes → antibodies -incubated for 60 minutes at 37°C → A liquid at 37°C for 30 minutes → Color liquid for 15 minutes → hematoxylin light stained for 1 minute → dehydration, transparent and neutral gum cementing. HMGA2-positive cells had nuclei containing brown granules, and CD9-positive cells had cell membrane and (or) cytoplasm containing brown granules, sections were randomly observed, and cases with an average rate of positive cells ≥25% were positive, and cases with an average rate of positive cells <25% were negative [16](Figures 1, 2, 3 and 4). The primary antibodies were replaced with 0.01 mol/L PBS solution (pH7.4). Effective gallbladder adenocarcinoma sections were positive controls, and the 0.01 mol/L PBS solution (pH7.4) was the negative control.

### Statistical analysis

All the experimental data were input using the SPPSS13.0 statistical package from SPSS Inc (Chicago,USA). The relationship between HMGA2 or CD9 expression and histological or clinical factors was analyzed by means of the χ^2^ test or Fisher's exact test, and a P-value <0.05 was statistically significant. The Kaplan-Meier method was used for univariate survival analysis (log-rank test), the Cox proportional hazards model was used for multivariate analysis, and Wald’s test was used to determine the 95% confidence interval.

## Results

### HMGA2 and CD9 expression in the gallbladder of benign and malignant lesions

HMGA2 immunohistochemical positive reaction product was mainly localized in the nucleus (Figure 1). CD9 immunohistochemical positive reaction product was localized in the cytoplasm and/or cell membrane (Figure 2). As shown in Table 1, HMGA2 expression of gallbladder adenocarcinoma was significantly higher than that of adjacent tissue, polyps and chronic cholecystitis gallbladder epithelium (*P* <0.01), but CD9 expression was the opposite (*P* <0.05 or *P* <0.01). The gallbladder epithelium of benign gallbladder diseases with HMGA2-positive and CD9-negative expression appeared to be from moderate to severe dysplasia.

**Table 1:** HMGA2 and CD9 expression in the gallbladder expression of benign and malignant lesions

**Type**	**Case Number**	**HMGA2**			**CD9**		
		**Positive Number (%)**	**χ2**	***P***	**Positive Number (%)**	**χ2**	***P***
Gallbladder adenocarcinoma	108	64(59.3)			57 (52.8)		
Cancer adjacency tissues	46	11(23.9)	16.13	<0.01	36 (78.3)	8.76	<0.01
polyps	15	3(20.0)	8.19	<0.01	12 (80.0)	3.96	<0.05
Chronic cholecystitis	35	5(14.3)	21.41	<0.01	31 (88.6)	14.31	<0.01

### The relationship between HMGA2 and CD9 expression and clinicopathological features of gallbladder cancer

The positive rates of HMGA2 were significantly lower in the cases of well-differentiated adenocarcinoma with a maximal diameter of mass <2 cm, no-metastasis of lymph node, and no-invasiveness of regional tissues than those of poorly-differentiated adenocarcinoma, maximal diameter of mass >2 cm, metastasis of lymph nodes, and invasiveness of regional tissues in gallbladder adenocarcinoma (*P* <0.05 or *P* <0.01), but the CD9 expression was the opposite (*P* <0.05 or *P* <0.01). There was no significant relationship between HMGA2 and CD9 expression and other clinical and pathological features of gallbladder cancer (*P* >0.05, Table 2).

**Table 2:** The relationship between HMGA2 and CD9 expression and clinicopathological features of gallbladder cancer

**Clinical and pathological features**	**Case Number**	**HMGA2**			**CD9**		
		**Positive Number(%)**	***χ***^**2**^	**P**	**Positive Number(%)**	***χ***^**2**^	**P**
Gender							
Male	24	15(62.5)	0.13	>0.05	11 (45.8)		
Female	84	49(58.3)			46 (54.8)	0.60	>0.05
Age (years)							
≤45	31	16(51.6)	1.05	>0.05	15 (48.4)	0.34	>0.05
>45	77	48(62.3)			42 (54.5)		
Pathological types *							
Well differentiated	36	12(33.3)	16.38	<0.01	26 (72.2)	10.26	<0.05
Differentiated	31	21(67.7)			15 (48.4)		
Poorly differentiated	30	24(80.0)			10 (33.3)		
Mucinous adenocarcinoma	11	7(63.6)			6 (54.5)		
Tumor maximum diameter (cm)							
<2	31	14(45.2)	3.70	<0.05	22 (71.0)	5.77	<0.05
≥2	77	50(64.9)			35 (48.6)		
Lymph node metastasis							
No	49	22(44.9)	7.66	<0.01	34 (69.4)	9.93	<0.01
Yes	59	42(61.2)			23 (39.0)		
Violations of the surrounding tissues							
No	49	21(42.9)	9.99	<0.01	33 (67.3)	7.64	<0.01
Yes	59	43(62.9)			24 (40.7)		
Gallbladder stones							
No	50	25(50.0)	3.31	>0.05	24 (48.0)	0.85	>0.05
Yes	58	39(67.2)			33 (56.9)		

### The relationship between HMGA2 and/or CD9 expression and survival time of patients with gallbladder cancer

The data from 67 cases of 108 cases of gallbladder adenocarcinoma were obtained by letter or telephone interviews. These included 20 cases with a survival time of ≥1-year postoperative, and 47 cases with a survival time of <1 year postoperative; the average survival period of 67 cases was 9.6 ± 5.2 months. By means of immunohistochemistry, 35 (52.2%) cases were shown to have HMGA2 positive expression and 32 cases (47.8%) to have CD9 expression. As shown in Figures 3 and 4, the Kaplan-Meier survival analysis suggests that after surgery the average survival time of gallbladder cancer patients was closely related to histological types (*P* = 0.031), maximum tumor diameter (*P* = 0.003), lymph node metastasis (*P* = 0.005) and invasion of surrounding tissue status (*P* = 0.002); the survival time of patients with HMGA2-positive expression was significantly lower than that of patients with HMGA2-negative expression (*P* = 0.020), and the survival time of patients with CD9-positive expression was significantly higher than that of patients with CD9-negative expression (*P* = 0.019). Cox multivariate analysis showed that the largest tumor diameter was ≥2 cm, that lymph node metastasis had occurred, invading the surrounding tissues and organs, and that HMGA2-positive expression or CD9-negative expression was negatively correlated with the postoperative survival time of patients. Also HMGA2-positive expression or CD9-negative expression were positively correlated with mortality of patients, were risk factors, and were independent prognostic factors; according to the relative degree of risk, HMGA2 had the greatest impact on prognosis (Table 3).

**Table 3:** Multivariate Cox regression analysis of survival ratio of 67 patients with gallbladder cancer

**Group**	**Factors**	**Regression coefficient**	**Standard error**	**Relative risk**	***P-*value**	**95% confidence interval**	
						**Lower limit**	**Upper limit**
Pathological types	Adenoma / well-differentiated - / moderately differentiated - / poorly differentiated / mucinous adenocarcinoma	0.549	0.302	1.73	0.068	0.96	3.13
Tumor diameter (cm)	<2.0/≥2.0	0.956	0.332	2.6	0.036	1.36	4.99
Lymph node metastasis	No/Yes	1.011	0.394	2.75	0.033	1.27	5.95
Violation of the surrounding tissues	No/Yes	1.025	0.384	2.79	0.023	1.31	5.92
HMGA2	-/+	1.105	0.331	3.02	0.018	1.58	5.78
CD9	-/+	−0.789	0.321	0.45	0.015	0.24	0.85

## Discussion

The HMGA2 protein was found in the late 1980s, as one of the high mobility protein family members, encoded by the HMGA2 gene which is located on chromosome 12 q14, 15, and has a molecular weight of approximately 12 KD. HMGA2 has complex functions, and the current study focuses on its relationship with cancer. Previous studies have shown that HMGA2 gene expression in adult tissues was very low or had no expression, and was highly expressed in the early embryo and the epithelial or mesenchymal origin of malignant tumors, suggesting that the HMGA2 gene plays an important role in the growth of higher eukaryotes and in the proliferation and differentiation of malignant cells [17,18]. Recently, some studies have shown that HMGA2 expression was closely related to the progression, invasion, metastasis and prognosis of a number of malignant tumors, and tumors with high HMGA2expression were highly malignant, and prone to invasive metastasis and poor prognosis [3-8]. However, there have not been reports about HMGA2 expression in the benign and malignant lesions of the gallbladder. Our data show that the HMGA2 positive expression ratio of gallbladder adenocarcinoma was significantly higher than that of the adjacent tissues, adenomatous polyps and gallbladder epithelium of chronic cholecystitis. Benign gallbladder epitheliums with HMGA2 positive expression appear to have moderate to severe dysplasia. The HMGA2 positive expression ratio of cases with well-differentiated adenocarcinoma, with the largest tumor diameter <2 cm, lymph node without metastasis and with organs which did not invade surrounding tissues was significantly lower than that of cases with poorly differentiated adenocarcinoma, the largest tumor diameter ≥2 cm, lymph node with metastasis and organs invading surrounding tissues. The Kaplan-Meier univariate survival analysis showed that after surgery, the survival time of cases with HMGA2-positive expression was significantly lower than that of cases with HMGA2-negative expression. The Cox multivariate analysis showed that HMGA2 expression was negatively correlated with the postoperative survival ratio, and was positively correlated with postoperative mortality, and was the evaluation factor for independent poor prognosis. These results suggest that the HMGA2 expression level of gallbladder adenocarcinoma was similar to that of other epithelial malignant tumors. HMGA2 also reflected progression and clinical behaviors of gallbladder cancer and was an important biological marker of prognosis. A high level of expression of HMGA2 showed poor prognosis.

CD9, also known as migration-associated protein −1 (MRP1), is a cell surface glycoprotein inhibiting cell movement, and belongs to the transmembrane 4 superfamily (TM4SF). After transfecting into cultured cells of non-hematopoietic cells, CD9 could inhibit the cell movement and, moreover, its inhibition is directly related with the quantity of CD9 expression, suggesting that CD9 was closely related with cell proliferation, cell motility, cell adhesion, invasion and metastasis of tumors [10]. Recent studies on CD9 expression of lung, breast, pancreatic and colon cancers and other malignant tumors and its clinicopathological significance, suggested that CD9 expression was related to tumor metastasis, could inhibit tumor metastasis effect, and, that when the tumor had lymph node or hematogenous metastasis, the CD9 expression level was significantly reduced [9-15,19]. Here, we investigated CD9 expression in the benign and malignant lesions of the gallbladder. Our data showed that the CD9-positive expression ratio of gallbladder cancer was significantly lower than that of the adjacent tissues, adenomatous polyps and gallbladder epithelium of chronic cholecystitis. The benign gallbladder epithelium CD9-negative expression showed moderate to severe dysplasia. The CD9 positive expression ratio of cases with well-differentiated adenocarcinoma, the largest tumor diameter <2 cm, lymph node without metastasis and organs, which did not invade surrounding tissues, was significantly higher than that of cases with the poorly differentiated adenocarcinoma, the largest tumor diameter ≥2 cm, lymph node with metastasis and organs invading surrounding tissues.

The Kaplan-Meier univariate survival analysis showed that after surgery the survival time of cases with CD9-positive expression was significantly higher than that of cases with CD9-negative expression. The Cox multivariate analysis showed that CD9 positive expression was positively correlated with the postoperative survival ratio, and was negatively correlated with postoperative mortality, and was the evaluation factor for independent poor prognosis. These results suggested that the CD9 expression level of gallbladder adenocarcinoma was similar to that of other epithelial malignant tumors. CD9 also reflected progression and clinical behaviors of the gallbladder cancer and was the important biological marker of prognosis. A high level expression of CD9 showed poor prognosis.

## Conclusions

In the present study, our data showed that the expression of HMGA2 and/or CD9 might be closely related to carcinogenesis, clinical biological behaviors, and prognosis of gallbladder adenocarcinoma. The data also suggest the prognostic significance of HMGA2 and/or CD9 expression as a tumor biological marker in patients with gallbladder adenocarcinoma.

## Competing interests

The authors declare that they have no competing interests.

## Authors’ contributions

QZ and ZY with Designing experiments, statistical analysis and writing manuscript; LX with doing experiments; FL with following-up patients and do part of experiments; LY and XM with collecting specimens and doing part of statistical analysis. All authors read and approved the final manuscript.
